# Accelerometer-based vibration analysis and oxygenator thrombosis in venovenous ECMO: an experimental porcine model

**DOI:** 10.1186/s40635-025-00763-7

**Published:** 2025-06-02

**Authors:** Lars Prag Antonsen, Svein Aslak Landsverk, Per Steinar Halvorsen, Amrit Thiara, Didrik Lilja, Naimahmed Nesaragi, Andreas Espinoza

**Affiliations:** 1 Department of Anesthesia and Intensive Care, Rikshospitalet, Oslo University Hospital, Sognsvannsveien 20, 0372 Oslo, Norway; 2 Department of Anesthesia and Intensive Care, Østfold Hospital Trust, Kalnesveien 300, 1714 Grålum, Norway; 3 Department of Research, Østfold Hospital Trust, Kalnesveien 300, 1714 Grålum, Norway; 4 The Intervention Centre, Oslo University Hospital, Sognsvannsveien 20, 0372 Oslo, Norway; 5 Faculty of Medicine, University of Oslo, Problemveien 11, 0313 Oslo, Norway; 6 Department of Anesthesia and Intensive Care, Ullevaal Hospital, Oslo University Hospital, Kirkeveien 166, 0450 Oslo, Norway; 7 Department of Cardiothoracic Surgery, Rikshospitalet, Oslo University Hospital, Sognsvannsveien 20, 0372 Oslo, Norway

**Keywords:** VV ECMO, Oxygenator thrombosis, Accelerometer-based vibration analysis

## Abstract

**Background:**

Oxygenator thrombosis is a potentially life-threatening complication during venovenous extracorporeal membrane oxygenation (VV ECMO). It can cause blood flow obstruction, impaired gas exchange, hematologic abnormalities, or sudden ECMO flow cessation. Early detection and timely circuit exchange is critical yet challenging. Acute clot formation necessitates immediate circuit replacement, while premature replacement risks unnecessary procedural harm and increased costs. No reliable method exists to detect early oxygenator thrombosis. Strategies include visual inspection, monitoring the pressure difference across the oxygenator (Δ*P*_oxy_), gas exchange evaluation, and blood tests. In the present animal study, we aimed to evaluate the feasibility of accelerometer-based vibration analysis as a real-time and non-invasive method for detecting oxygenator thrombosis during VV ECMO. We hypothesized that accelerometer signals would change concurrently with or precede increases in Δ*P*_oxy_.

**Methods:**

The study was performed on anesthetized and mechanically ventilated pigs (*n* = 7) on VV ECMO. Hemodynamic parameters, ECMO circuit pressures, and signals from an accelerometer attached to the ECMO oxygenator were continuously recorded at different pump speeds, and after anticoagulation reversal to promote thrombosis within the ECMO oxygenator.

**Results:**

The primary finding of this study was a significant increase in the accelerometer signal's Root Mean Squared (RMS_oxy_) 15 min after anticoagulation reversal, with no rpm adjustment and without corresponding changes in Δ*P*_oxy_. Variations in RMS_oxy_ associated with high ECMO pump speed and circuit flow were discernible from those observed following anticoagulation reversal.

**Conclusion:**

The present animal study demonstrates the feasibility of accelerometer-based vibration analysis as a real-time and non-invasive method for detecting vibrations associated with reversal of anticoagulation and potential oxygenator thrombosis during VV ECMO.

**Supplementary Information:**

The online version contains supplementary material available at 10.1186/s40635-025-00763-7.

## Introduction

Oxygenator thrombosis is a common and potentially life-threatening complication during venovenous extracorporeal membrane oxygenation (VV ECMO). Oxygenator thrombosis and/or circuit clotting is reported to occur in 5–30% of patients and can lead to blood flow obstruction, compromised gas exchange capacity, circuit-related hematologic abnormalities including hemolysis, disseminated intravascular coagulation (DIC), bleeding and sudden ECMO flow cessation [1–8].

Early identification of oxygenator thrombosis and optimal timing for ECMO circuit exchange remains a significant challenge [9]. Acute clot formation represents an emergency, requiring immediate circuit exchange to prevent potentially life-threatening complications. However, premature replacement of an adequately functioning circuit exposes the patient to unnecessary procedural risks and to increased costs [9–11].

Currently, no reliable method exists to detect oxygenator thrombosis in its early stages. Common strategies include monitoring the pressure difference across the oxygenator (Δ*P*_oxy_), visual inspection of the oxygenator, evaluating gas exchange efficiency, and analyzing blood tests such as D-dimers, platelets, free hemoglobin (fHb), lactate dehydrogenase (LDH) and fibrinogen. Additionally, coagulation tests such as activated partial thromboplastin time (aPTT), activated clotting time (ACT), prothrombin time international normalized ratio (PT/INR), anti-factor Xa, thromboelastography (TEG) and rotational thromboelastometry (ROTEM) are used as indirect indicators of oxygenator/circuit clotting [9, 12]. All these methods have limitations.

An ideal detection method for oxygenator thrombosis should be non-invasive, accurate, and capable of real-time monitoring. It should be able to assess the oxygenator directly, independently of coagulation status, as normal hemostasis is difficult to achieve in patients on ECMO. No such method currently exists [9].

Accelerometer-based vibration analysis is a well-established technology used to detect mechanical failures across various industries. It is commonly used to monitor and diagnose issues in engines, pumps, machinery as well as in medical devices [13–16]. It has recently been shown that accelerometers can detect subtle vibrations associated with turbulence and thrombosis [15–20] in left ventricular assist devices (LVAD), but it has not yet been applied to ECMO.

In the present animal study, we aimed to evaluate the feasibility of accelerometer-based vibration analysis as a real-time and non-invasive method for detecting oxygenator thrombosis during VV ECMO. We hypothesized that accelerometer signals would change concurrently with or precede increases in Δ*P*_oxy_.

## Methods

The experimental protocol is part of a larger study investigating various aspects of ECMO-related complications and their detection [21]. We conducted eight pilot experiments to plan the study and performed the protocol in ten healthy Noroc pigs of both genders. Three pigs were excluded from the analysis: two died before the experiment was completed, and one was excluded due to equipment malfunction. Randomization and blinding were not applicable in this study.

The median weight was 61.5 kg (58 kg–67 kg). The study protocol was approved by the Norwegian National Animal Research Authority (trial registration number 24306 and 28,798) and was performed in accordance with European legislation and the ARRIVE 2.0 guidelines for animal research [22], carefully adhering to the three R's of animal research: replacement by minimizing animal use, reduction through careful experimental design, and refinement by optimizing procedures to reduce animal discomfort [23]. A porcine model was chosen because of the similarities to human cardiac anatomy and physiology [24].

The pigs were subjected to fasting overnight with free access to water in an animal research facility, and premedicated by intramuscular injection of 30 mL ketamine 50 mg/mL (~ 25 mg/kg), 4 mL azaperone 40 mg/mL (~ 2.5 mg/kg) and 1 mL atropine 1 mg/mL (~ 15 μg/kg). Anesthesia was maintained with pentobarbital 4 mg/kg/h, morphine 2 mg/kg/h and midazolam 0.15 mg/kg/h. Ringer’s acetate solution was infused at 10 mL/kg/h until the start of the interventions. Total blood volume was estimated to be ~ 60 mL/kg (~ 3600 mL).

Electrocardiography (ECG), peripheral oxygen saturation (S_p_O_2_), bladder temperature, invasive arterial pressure, central venous pressure (CVP) and pressures from the inflow and outflow limbs of the ECMO circuit were obtained using standard bedside monitors (Life Scope^®^ Monitor, Nihon Kohden, Japan). A pulmonary artery (PA) catheter and a central venous line (CVL) were inserted via the internal jugular veins. PA pressure was measured with a PA catheter (Swan-Ganz CCOmbo, Edwards Lifesciences, Irvine, CA). All pressures were zeroed to atmospheric pressure and calibrated according to manufacturer’s specifications.

The animals were mechanically ventilated via a tracheostomy with tidal volume 4–5 mL/kg, respiratory rate (RR) 16–18/min, positive end-expiratory pressure (PEEP) 5 cmH₂O and inspired oxygen fraction (F_i_O_2_) 0.5. No changes were made during registration.

The ECMO return cannula (17 French, Bio-Medicus^™^, Medtronic Inc., Fridley, United States) was inserted in the right external jugular vein with the cannula tip in the right atrium. The drainage cannula (23 French, Maquet/Getinge AB, Göteborg, Sweden) was inserted via the right femoral vein with the cannula tip in the inferior vena cava (IVC). The cannulas were inserted percutaneously ultrasound-guided using Seldinger’s technique during fluoroscopy to ensure proper positioning. To prevent ECMO circuit clotting, a bolus of intravenous (iv) heparin 2 mg/kg followed by an infusion of 0.5 mg/kg/h to obtain an activated clotting time (ACT) of 180–240 s was given. The ECMO circuit was run in the femoro-atrial direction by a standard ECMO machine and oxygenator (Cardiohelp/HLS Set Advanced 7.0, Maquet/Getinge AB, Göteborg, Sweden).

### Signal acquisition and analysis

**Fig. 1:**
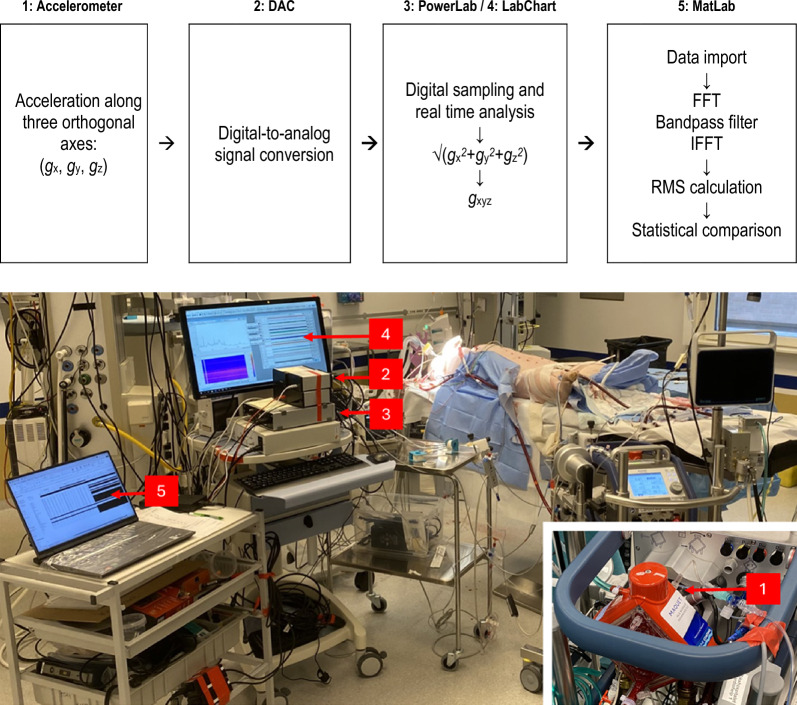
Signal acquisition and analysis. 1: Accelerometer and oxygenator. 2: DAC: digital-to-analog converter. 3: PowerLab. 4: LabChart. 5: MatLab. *g*_x_, *g*_y_*, g*_z_: accelerometer signals. FFT: fast Fourier transform. IFFT: Inverse fast Fourier transform. RMS: root mean square

The signal acquisition and analysis process is presented in Fig. 1. A triaxial accelerometer (CS1^®^, Cardiaccs AS, Oslo, Norway) capable of measuring acceleration along three orthogonal axes was mounted on the ECMO oxygenator. The signals from each axis (*g*_x_, *g*_y_*, g*_z_) were processed through a digital-to-analog converter (CB1 Research DAC, Cardiaccs AS, Oslo, Norway) before being collected in a digital acquisition system (PowerLab 16/35 and LabChart 8, ADInstruments, Auckland, NZ). We computed an omnidirectional representation of acceleration *g*_xyz_ as the norm √ (*g*_x_^2^ + *g*_y_^2^ + *g*_z_^2^). Signal processing and analysis were performed in MATLAB (R2022b, The MathWorks Inc., Natick, MA) and involved multiple stages, including bandpass filtering, root mean square (RMS) calculations, and statistical analysis.

The initial step was to transform the time domain signal to the frequency domain using the Fast Fourier Transform (FFT). The FFT allows the decomposition of the signal into its frequency components, making it easier to manipulate specific frequency ranges. A frequency-domain bandpass filter was applied to retain frequencies between 10 and 375 Hz by setting the corresponding Fourier coefficients to zero. This frequency-domain approach approximates a bandpass filter with sharp cutoffs. After filtering, the signal was transformed back to the time domain using the Inverse Fast Fourier Transform (IFFT). The IFFT was performed with symmetric reconstruction to ensure that no complex components were introduced into the signal.

The filtered signal was processed using an RMS calculation with a sliding non-overlapping window of 30 s. Key metrics, including RMS_oxy_, Δ*P*_oxy_, and hemodynamic parameters, were recorded continuously and extracted at specific time intervals specified below. Due to the small sample size, non-parametric statistical methods were employed. The data were analyzed as paired measurements and compared using the Wilcoxon signed-rank test. Significance level was set at p ≤ 0.05.

### Experimental protocol

**Fig. 2:**
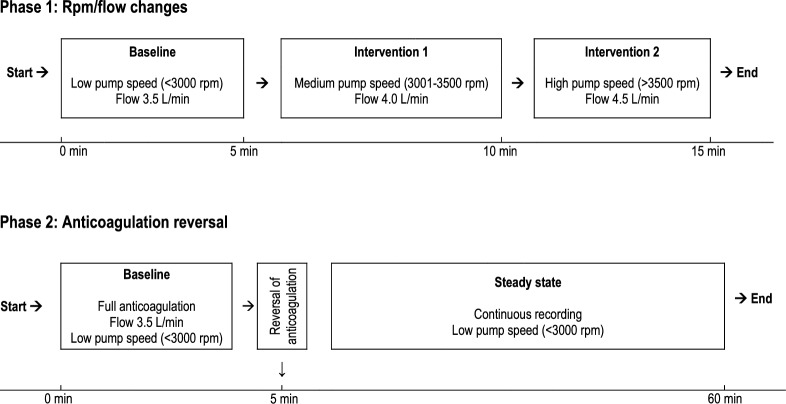
Timeline of interventions. Phase 1: Three different pump speeds: low (< 3000 rpm), medium (3001-3500 rpm) and high (> 3500 rpm). Phase 2: Reversal of anticoagulation

The experimental protocol consisted of two main phases and is presented in Fig. 2. The first phase involved obtaining data at three different pump speeds: low (< 3000 rpm), medium (3001–3500 rpm) and high (> 3500 rpm). The second phase focused on reversing anticoagulation and continuously monitoring the system's response over a 60-min period.

The first phase began by adjusting the ECMO pump speed to achieve a flow rate of 3.5 L/min. Once steady-state flow was established, baseline data were recorded. The flow rate was then increased to 4.0 L/min by adjusting rpm, and another set of measurements was collected under steady-state conditions. Finally, the flow rate was increased to 4.5 L/min, and a third set of measurements was obtained.

In the second phase, anticoagulation was reversed by administering protamine iv at a dose of 2 mg/kg, along with 2000 mg of tranexamic acid iv and 5 mmol of calcium chloride iv. Following the administration of these agents, the experimental setup was maintained for a total of 60 min to allow for continuous collection accelerometer signals, ECMO circuit pressures and hemodynamic parameters.

## Results

**Fig. 3:**
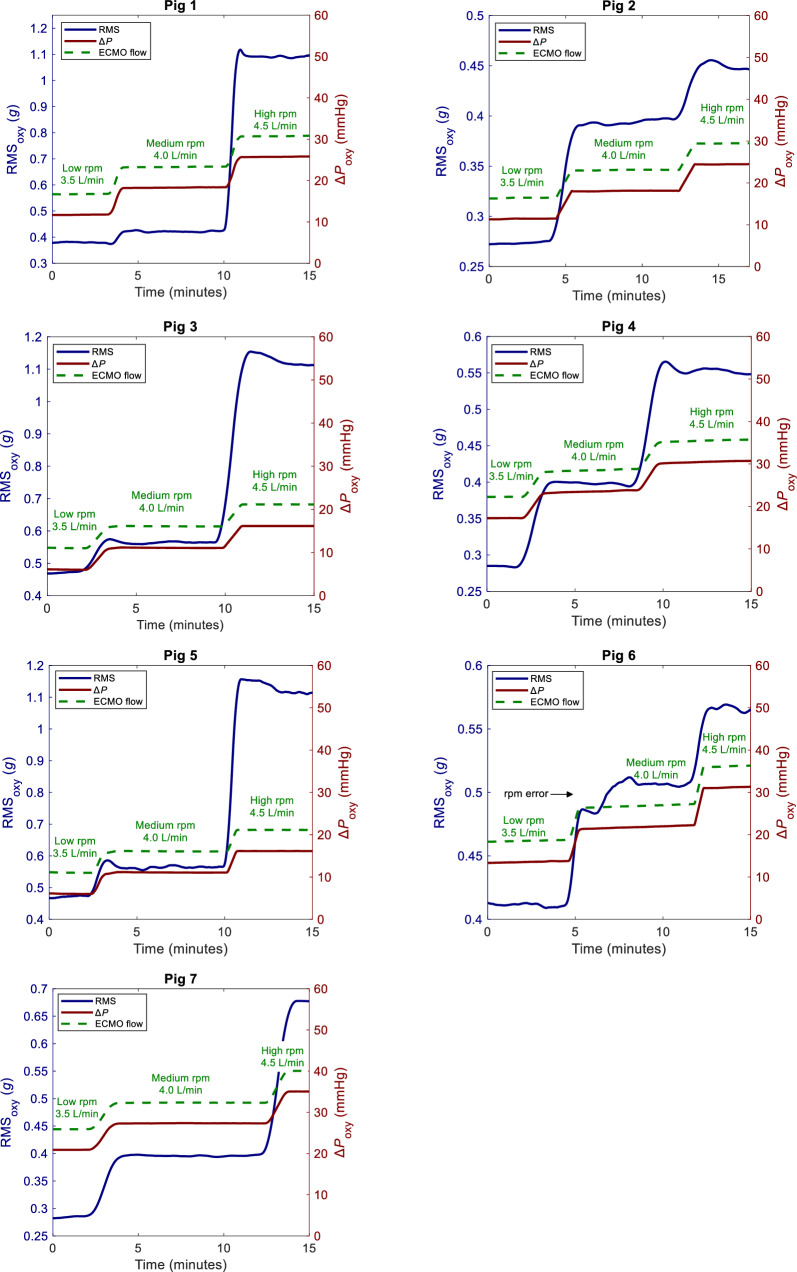
The impact of pump speed on RMS_oxy_ and Δ*P*_oxy_. RMS_oxy_: root mean squared of oxygenator vibration signal. Rpm: revolutions per minute. Δ*P*_oxy_: Oxygenator transmembrane pressure

**Fig. 4:**
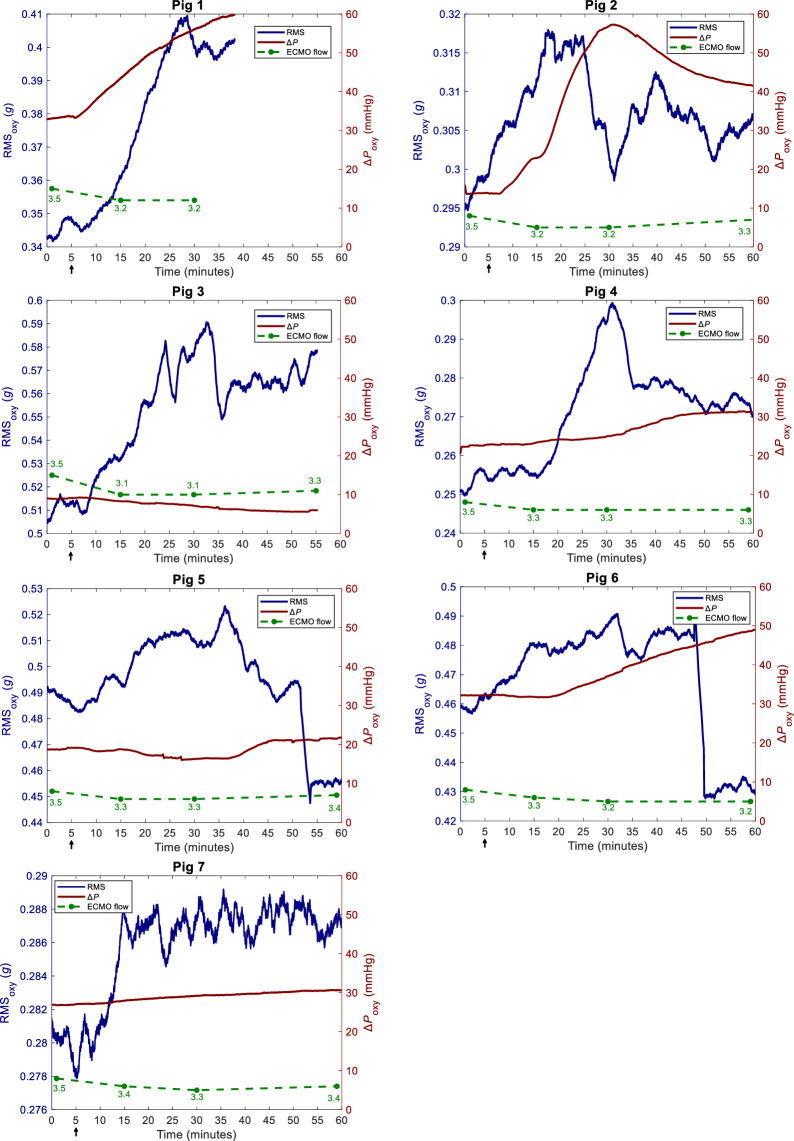
The impact of anticoagulation reversal on RMS_oxy_ and Δ*P*_oxy_. RMS_oxy_: root mean squared of oxygenator vibration signal. ∆*P*_oxy_: oxygenator transmembrane pressure. ECMO flow: L/min. Arrow indicates time of anticoagulation reversal. In Pig 1 and Pig 3, the registration time is shorter due to equipment malfunction

The results of this study are presented in Figs. 3, 4 and Table 1. The key findings are (1) a significant increase in RMS_oxy_ observed 15 min following anticoagulation reversal with no rpm adjustment and without corresponding changes in Δ*P*_oxy_, and (2) that the changes in RMS_oxy_ related to high ECMO pump speeds were larger than the variations observed after anticoagulation reversal. The magnitude and durability of the changes varied across animals. Importantly, RMS_oxy_ and Δ*P*_oxy_ remained stable within each pump speed level.

**Table 1:** ECMO circuit data,_,_hemodynamics and RMS

Pump speed					
	Baseline	Medium rpm	*p*	High rpm	*p*
SpO_2_ (%)	100 (93—100)	100 (90—100)	0.71	100 (90—100)	0.41
HR (bpm)	80 (52—157)	90 (52—180)	**0.04**	89 (52—202)	**0.04**
MAP (mmHg)	79 (70—100)	73 (67—104)	0.20	73 (65—105)	0.67
CVP (mmHg)	7 (-1—11)	7 (-2—11)	1.00	7 (-1—12)	0.08
MPAP (mmHg)	19 (16—22)	20 (16—22)	0.71	20 (16—23)	0.59
ACT (s)	232 (191—293)	232 (191—293)	1.00	232 (191—293)	1.00
ECMO Flow (L/min)	3.5 (3.5 – 3.5)	4.0 (4.0 – 4.0)	**0.01**	4.5 (4.5 – 4.5)	**0.01**
Δ*P*_oxy_ (mmHg)	12 (6—21)	18 (11—27)	**0.02**	26 (16—35)	**0.02**
RMS_oxy_ (*g*)	0.381 (0.273—0.474)	0.421 (0.392—0.566)	**0.02**	0.677 (0.455—1.113)	**0.02**
∆RMS_oxy_ (%)	N/A	23.1 (10.6 – 43.5)	**0.02**	135.0 (38.3 – 186.6)	**0.02**

Anticoagulation reversal							
	Baseline	15 min	*p*	30 min	*p*	60 min	*p*
SpO_**2**_ (%)	100 (99—100)	100 (99—100)	1.00	100 (99—100)	1.00	100 (99—100)	1.00
HR (bpm)	85 (52—157)	85 (53—112)	0.33	84 (54—115)	0.34	98 (59—115)	**0.02**
MAP (mmHg)	73 (65—88)	72 (65—85)	**0.04**	72 (65—81)	**0.04**	72 (59—81)	**0.03**
CVP (mmHg)	6 (3—9)	6 (3—10)	0.56	7 (3—10)	0.18	6 (3—8)	0.41
MPAP (mmHg)	21 (15—22)	21 (16—22)	0.26	20 (16—22)	0.44	20 (16—23)	0.79
ACT (s)	220 (191—252)	175 (150—220)	**0.02**	125 (109—145)	**0.02**	117 (100—130)	**0.02**
ECMO Flow (L/min)	3.5 (3.5—3.5)	3.3 (3.1—3.4)	**0.02**	3.2 (3.1—3.3)	**0.02**	3.3 (3.2—3.4)	**0.02**
Δ*P*_oxy_ (mmHg)	22 (9—33)	28 (8—49)	0.25	29 (7—57)	0.13	31 (6—56)	**0.04**
RMS_oxy_ (*g*)	0.343 (0.251- 0.506)	0.385 (0.266—0.556)	**0.02**	0.399 (0.288—0.581)	**0.02**	0.399 (0.269—0.573)	0.40
∆RMS_oxy_ (%)	N/A	6.4 (2.4—12.3)	**0.02**	6.5 (1.7—18.6)	**0.02**	4.2 (-7.2- 16.3)	0.24

Data as median (min–max). Significant *p*-values in bold

*SpO*_*2*_ peripheral oxygen saturation, *HR* heart rate, *MAP* mean arterial pressure, *MPAP* mean pulmonary artery pressure, *CVP* central venous pressure, *ΔP*_*oxy*_*, RMS*_*oxy*_ Root Mean Square of oxygenator vibration signal, *∆RMS*_*oxy*_ Difference in Root Mean Square of the oxygenator vibration signal, *ACT* activated clotting time

At medium and high pump speed, RMS_oxy_, ΔRMS_oxy_, Δ*P*_oxy_ and HR increased. S_p_O_2,_ MAP, CVP, MPAP and ACT did not change significantly. Flow increased according to rpm adjustments in the experimental protocol. No clots were observed in the oxygenator or cannulas.

RMS_oxy_ and ΔRMS_oxy_ increased significantly at 15 and 30 min post-anticoagulation reversal but decreased by 60 min. Δ*P*_oxy_ increased significantly at 60 min but not at 15 and 30 min. HR remained unchanged at 15 and 30 min but increased at 60 min. MAP decreased by 15 min and then stabilized. S_p_O_2_, CVP, and MPAP remained stable throughout. ACT normalized by 15 min and stayed stable. ECMO flow decreased after 15 min and then stabilized. No clots were observed in the oxygenator or cannulas.

## Discussion

The primary finding in the present study is a small, but significant increase in RMS_oxy_ 15 min following anticoagulation reversal, with no rpm adjustment and without corresponding changes in Δ*P*_oxy_. It indicates that vibrations can be detected before a measurable rise in Δ*P*_oxy_.

Second, the changes in RMS_oxy_ related to ECMO pump speed were, as expected, larger than the variations observed after anticoagulation reversal. Importantly, RMS_oxy_, Δ*P*_oxy_, S_p_O₂, HR, MAP, MPAP and CVP remained stable within each pump speed level. These findings support the notion that vibrations due to changes in pump speed are discernible from subtle changes associated with anticoagulation reversal.

Third, RMS_oxy_ increased following anticoagulation reversal, despite a decrease in ECMO flow. In contrast, during rpm increase, both ECMO flow and RMS_oxy_ increased simultaneously. This may indicate that vibrations are not only related to measurable differences in ECMO flow and circuit pressures.

Last, the changes in RMS_oxy_ and Δ*P*_oxy_ after 60 min varied. None of the oxygenators clotted completely. It highlights that the progression of oxygenator thrombosis can be a prolonged process [25], and that variations in resistance, fluid properties and altered flow patterns contribute to variations in turbulence in flow systems like the ECMO oxygenator. These dynamics may partially explain the subsequent decrease in RMS_oxy_.

Currently, no reliable method exists for the early detection of oxygenator thrombosis, as all commonly used strategies have limitations.

A persistent or progressive increase in Δ*P*_oxy_ or a significant decrease in blood flow despite maintaining pump speed raises clinical suspicion for oxygenator thrombosis. However, an increase in Δ*P*_oxy_ due to thrombosis depends not only on the size of the clot, but also on its location in the oxygenator. Clots in low flow areas of the oxygenator cause less resistance than clots in high flow areas [26, 27]. Thus, changes in Δ*P*_oxy_ are not necessarily proportional to clot formation. Consequently, there is currently no consensus on a specific threshold that mandates circuit replacement [6, 9, 10, 27–31].

Visual inspection of the oxygenator reveals clot formation only in visible regions of the membrane, making it a late and unreliable indicator of thrombosis [6, 32]. Gas exchange efficiency correlates poorly with clot volume and the need for oxygenator exchange [27, 33].

Coagulation abnormalities in patients on ECMO have multiple potential causes beyond thrombosis. Elevated D-dimer is associated with DIC, trauma, and surgical interventions [6, 10, 34–37], while low fibrinogen is associated with DIC and liver disease [38, 39]. Altered platelet count is influenced by transfusions, DIC and infection [40, 41], and Anti-factor Xa is impacted by hemolysis and antithrombin deficiency [42, 43]. Increased fHb and LDH levels may indicate hemolysis due to thrombotic events but are also linked to blood product administration and high pump speeds that cause mechanical damage to blood cells [44]. The ability of TEG, ROTEM, PT/INR, aPTT and ACT to predict ongoing thrombosis remains unclear [45–49].

Real-time oxygenator monitoring would enhance clinical decision-making. Several experimental methods have been proposed, but none of them have so far proven superior accuracy. They include ultrasound-based techniques [26], shunt flow monitoring [31, 50], CO_2_ exhaust gas monitoring [27, 51], indocyanine green (ICG) fluorescence imaging [52], infrasound detection [25], computer tomography (CT) scanning [53], micro-optical sensors [54] and pressure fluctuation analysis [9].

It has recently been shown that accelerometers can detect vibrations associated with turbulence and thrombosis [15–20] in LVAD, but it has not yet been applied to ECMO.

In the present study, changes in RMS_oxy_ preceded increases in Δ*P*_oxy_. RMS is a commonly used metric in vibration analysis that quantifies signal energy by considering both amplitude and duration. RMS is suitable for continuous vibration analysis as it is sensitive to high-amplitude fluctuations, scale-independent, robust against noise and easily comparable across time points. We propose the possibility of using accelerometer-based vibration analysis as a real-time and non-invasive method for detecting vibrations associated with reversal of anticoagulation and potential oxygenator thrombosis during VV ECMO.

## Limitations

This study has several limitations. First, the registration period was relatively short and none of the oxygenators clotted completely. Second, the sample size was small (*n* = 7). Third, the ECMO circuit may be influenced by recirculation [21] and microbubbles, as this study is part of a larger project investigating ECMO complications. Last, there were considerable variations in hemodynamic data, ECMO circuit pressures, and accelerometer signals. Future studies could aim to evaluate vibration analysis through comparative analyses, alternative prothrombotic strategies such as clot infusion, administration of coagulation factors and/or longer observation periods.

## Conclusion

The present animal study demonstrates the feasibility of accelerometer-based vibration analysis as a real-time and non-invasive method for detecting vibrations associated with reversal of anticoagulation and potential oxygenator thrombosis during VV ECMO.

## Supplementary Information

Supplementary material 1.

## Data Availability

The datasets generated and analyzed during the current study are available from the corresponding author on reasonable request.

## Abbreviations

ACT: Activated clotting time
APTT: Activated partial thromboplastin time
CO: Cardiac output
CT: Computer tomography
CVL: Central venous line
CVP: Central venous pressure
DIC: Disseminated intravascular coagulation
ECG: Electrocardiography
FFT: Fast Fourier Transform
fHb: Free hemoglobin
F_i_O_2_: Inspired oxygen fraction
HR: Heart rate
ICG: Indocyanine green
IVC: Inferior vena cava
LDH: Lactate dehydrogenase
LVAD: Left ventricular assist device
MAP: Mean arterial pressure
MPAP: Mean pulmonary arterial pressure
PA: Pulmonary artery
P_a_O_2_: Arterial oxygen partial pressure
PEEP: Positive end-expiratory pressure
PT/INR: Prothrombin time, international normalized ratio
ROTEM: Rotational thromboelastometry
RMS_oxy_: Root mean squared of oxygenator vibration signal
RR: Respiratory rate
S_p_O_2_: Peripheral oxygen saturation
SVC: Superior vena cava
TEG: Thromboelastography
VV ECMO: Venovenous extracorporeal membrane oxygenation
Δ*P*_oxy_: Change in oxygenator transmembrane pressure
∆RMS_oxy_: Change in Root mean squared of oxygenator vibration signal
