# Supplementary material for: Accelerometer-based vibration analysis and oxygenator thrombosis in venovenous ECMO: an experimental porcine model
Source: Intensive Care Med Exp. 2025 Jun 2;13:55. doi: 10.1186/s40635-025-00763-7 (PMC12130417; doi:10.1186/s40635-025-00763-7)
Supplement: Supplementary file 1 — Supplementary material 1. [file 40635_2025_763_MOESM1_ESM.docx]

**ARRIVE 2.0 Checklist For Animal Research**

**Study design**

The study is an experimental porcine model using anesthetized and mechanically ventilated pigs to investigate accelerometer-based vibration analysis for detection of oxygenator thrombosis during venovenous ECMO. The study protocol was approved by the Norwegian National Animal Research Authority (trial registration number 24306 and 28798). A porcine model was chosen because of the similarities to human cardiac anatomy and physiology.

**Sample size and inclusion/exclusion criteria**

The study was performed using a total of 18 Noroc pigs of either gender. We conducted eight pilot experiments to plan the study and performed the experimental protocol in ten animals. Three pigs were excluded: two died before the protocol was completed, and one was excluded due to equipment malfunction. Seven pigs were included in the analyses.

**Randomization and blinding**

Randomization and blinding were not applicable in this study.

**Outcome measures and statistical methods**

We measured and assessed hemodynamic data (peripheral oxygen saturation, heart rate, arterial pressure, pulmonary artery pressure and central venous pressure), ECMO data (circuit flow and circuit pressures) and accelerometer data. To compare data across timepoints, we employed non-parametric statistical methods. The data were analyzed as paired measurements and compared using the Wilcoxon signed-rank test. Significance level was set at *p* ≤ 0.05.

**Experimental animals and procedures**

We performed the experimental protocol in ten Noroc pigs of either gender. The median weight was 61.5 kg (58 kg - 67 kg). The pigs were subjected to fasting overnight with free access to water in an animal research facility, and premedicated by intramuscular injection of 30 mL ketamine 50 mg/mL (~25 mg/kg), 4 mL azaperone 40 mg/mL (~2.5 mg/kg) and 1 mL atropine 1 mg/mL (~15 μg/ kg). Anesthesia was maintained with pentobarbital 4 mg/kg/h, morphine 2 mg/kg/h and midazolam 0.15 mg/kg/h. Ringer’s acetate solution was infused at 10 mL/kg/h until the start of the interventions. Total blood volume was estimated to be ~60 mL/kg (~3600 mL). The animals were mechanically ventilated via a tracheostomy with tidal volume 4-5 mL/kg, respiratory rate (RR) 16-18/min, positive end-expiratory pressure (PEEP) 5 cmH₂O and inspired oxygen fraction (F_i_O_2_) 0.5.

The ECMO return cannula was inserted in the right external jugular vein with the cannula tip in the right atrium. The drainage cannula was inserted via the right femoral vein with the cannula tip in the inferior vena cava. The cannulas were inserted percutaneously ultrasound-guided using Seldinger’s technique during fluoroscopy to ensure proper positioning. To prevent ECMO circuit clotting, a bolus of intravenous heparin 2 mg/kg followed by an infusion of 0.5 mg/kg/h to obtain an activated clotting time (ACT) of 180-240 seconds was given. The ECMO circuit was run in the femoro-atrial direction by a standard ECMO machine and oxygenator.

The experimental protocol consisted of two main phases. The first phase involved obtaining data at three different pump speeds: low (<3000 rpm), medium (3001-3500 rpm) and high (>3500 rpm). The second phase focused on reversing anticoagulation and continuously monitoring the system's response over a 60-minute period.

**Results**

The key findings of the present study are 1) a significant increase in the Root Mean Squared of the accelerometer signal from the ECMO oxygenator (RMS_oxy_) observed 15 minutes following anticoagulation reversal with no rpm adjustment and without corresponding changes in pressure difference across the oxygenator (ΔP_oxy_,) and 2) that the changes in RMS_oxy_ related to high ECMO pump speeds were larger than the variations observed after anticoagulation reversal. The magnitude and durability of the changes varied across animals.
